# *catena*-Poly[[[diaqua­(1,3-benzimidazole-κ*N* ^3^)manganese(II)]-μ-benzene-1,3-di­carboxyl­ato-κ^3^ *O* ^1^,*O* ^1′^:*O* ^3^] dihydrate]

**DOI:** 10.1107/S1600536811008555

**Published:** 2011-03-12

**Authors:** Xiao-Hui Wang

**Affiliations:** a Zhejiang College of Construction, Hangzhou 311231, People’s Republic of China

## Abstract

In the polymeric title complex, {[Mn(C_8_H_4_O_4_)(C_7_H_6_N_2_)(H_2_O)_2_]·2H_2_O}_*n*_, the Mn^II^ cation is coordinated by two benzene­dicarboxyl­ate anions, one benzimidazole ligand and two water mol­ecules in a distorted MnNO_5_ octa­hedral geometry. In the crystal, each benzene­dicarboxyl­ate anion bridges adjacent Mn^II^ cations through the terminal carboxyl­ate groups, forming a polymeric complex chain along the *a* axis. One Mn—O_carboxyl­ate_ bond is much longer than the others. In the crystal, π–π stacking is observed between nearly parallel [dihedral angle = 4.32 (6)°] benzimidazole aromatic ring systems of adjacent mol­ecules, the centroid–centroid distance between the imidazole and benzene rings being 3.5421 (11) Å. Extensive inter­molecular O—H⋯O and N—H⋯O hydrogen bonding is present in the crystal structure. The two lattice water mol­ecules are located on twofold rotation axes.

## Related literature

For background to π–π stacking, see: Deisenhofer & Michel (1989 ▶). For a related structure, see: Hu *et al.* (2006 ▶). For a longer Mn—O bond length in complex with a seven-coordinate Mn^II^ atom, see: Liu *et al.* (2005 ▶).
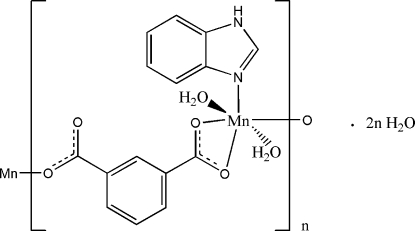


## Experimental

### 

#### Crystal data

[Mn(C_8_H_4_O_4_)(C_7_H_6_N_2_)(H_2_O)_2_]·2H_2_O
*M* *_r_* = 409.25
Orthorhombic, 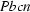

*a* = 18.9148 (12) Å
*b* = 23.7112 (16) Å
*c* = 7.8701 (4) Å
*V* = 3529.7 (4) Å^3^
*Z* = 8
Mo *K*α radiation
μ = 0.79 mm^−1^
*T* = 292 K
0.24 × 0.20 × 0.18 mm

#### Data collection

Rigaku R-AXIS RAPID IP diffractometer
Absorption correction: multi-scan (*ABSCOR*; Higashi, 1995 ▶) *T*_min_ = 0.786, *T*_max_ = 0.880
20574 measured reflections
4043 independent reflections
3261 reflections with *I* > 2σ(*I*)
*R*_int_ = 0.029

#### Refinement

*R*[*F* ^2^ > 2σ(*F* ^2^)] = 0.032
*wR*(*F* ^2^) = 0.091
*S* = 1.04
4043 reflections
236 parameters
H-atom parameters constrained
Δρ_max_ = 0.47 e Å^−3^
Δρ_min_ = −0.26 e Å^−3^

### 

Data collection: *PROCESS-AUTO* (Rigaku, 1998 ▶); cell refinement: *PROCESS-AUTO*; data reduction: *CrystalStructure* (Rigaku/MSC, 2002 ▶); program(s) used to solve structure: *SIR92* (Altomare *et al.*, 1993 ▶); program(s) used to refine structure: *SHELXL97* (Sheldrick, 2008 ▶); molecular graphics: *ORTEP-3* (Farrugia, 1997 ▶); software used to prepare material for publication: *WinGX* (Farrugia, 1999 ▶).

## Supplementary Material

Crystal structure: contains datablocks I, global. DOI: 10.1107/S1600536811008555/xu5169sup1.cif

Structure factors: contains datablocks I. DOI: 10.1107/S1600536811008555/xu5169Isup2.hkl

Additional supplementary materials:  crystallographic information; 3D view; checkCIF report

## Figures and Tables

**Table 1:** Selected bond lengths (Å)

Mn—N3	2.1657 (15)
Mn—O2^i^	2.0995 (13)
Mn—O3	2.1832 (13)
Mn—O4	2.6075 (16)
Mn—O5	2.1642 (14)
Mn—O6	2.1674 (14)

Symmetry code: (i) 
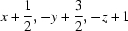
.

**Table 2:** Hydrogen-bond geometry (Å, °)

*D*—H⋯*A*	*D*—H	H⋯*A*	*D*⋯*A*	*D*—H⋯*A*
N1—H1⋯O9^ii^	0.86	1.94	2.795 (2)	171
O5—H5*A*⋯O4^iii^	0.89	1.89	2.7605 (18)	168
O5—H5*B*⋯O1^iv^	0.88	1.96	2.8120 (18)	164
O6—H6*A*⋯O4^v^	0.85	1.95	2.7553 (18)	159
O6—H6*B*⋯O1^vi^	0.86	1.88	2.7379 (18)	176
O7—H7*A*⋯O1	0.95	1.99	2.8844 (18)	155
O8—H8*A*⋯O3	0.94	1.79	2.7303 (18)	176
O9—H9*A*⋯O7	0.94	1.92	2.846 (2)	167
O9—H9*B*⋯O8	0.91	1.92	2.799 (2)	162

Symmetry codes: (ii) 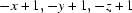; (iii) 
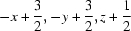
; (iv) 
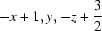
; (v) 
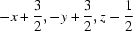
; (vi) 
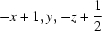
.

**Fig. 1.**
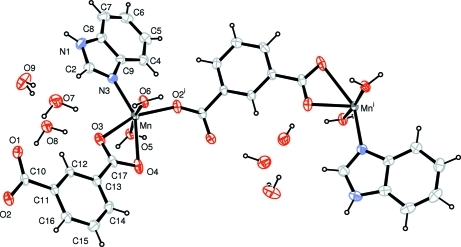
A fragment of the polymeric structure of (I) with 40% probability displacement ellipsoids (arbitrary spheres for H atoms) [symmetry code: (i) 1/2 + x,3/2 - y,1 - z].

**Fig. 2.**
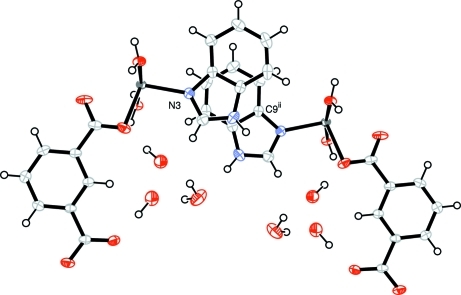
A diagram showing the partially overlapped arrangement between aromatic rings [symmetry code: (ii) x,1 - y,-1/2 + z].

## supplementary crystallographic information

### Comment

π-π Stacking between aromatic rings plays an important role in the electron transfer process in some biological system (Deisenhofer & Michel, 1989). Many structures of metal complexes incorporating aromatic ring ligands have been reported (Hu & Lai, 2006). The title Mn^II^ complex includes both of benzimidazole (bzim) and benzendicarboxylate (bdc) aromatic ring ligands, its crystal structure is presented here.

A fragment of the title polymeric Mn^II^ complex, together with lattice water molecules, is shown in Fig. 1. The Mn^II^ ion is surrounded by two bdc anions, one bzim and two coordination water molecules in a distorted octahedral geometry. Each bdc anion bridges two Mn^II^ ions to form the one dimensional polymeric chain running along the *a* axis. The Mn—O4 distance of 2.6075 (16) Å is much longer than the Mn—O3 bond distance (Table 1) but is comparable to 2.5356 (16) Å found in a seven-coordination Mn^II^ complex (Liu *et al.* 2005).

In the crystal structure, the partially overlapped arrangement is observed between nearly parallel bzim ring systems [dihedral angle 4.32 (6)°] (Fig. 2); the centroid-to-centroid separation between the N3-imidazole and C9^i^-benzene rings is 3.5421 (11) Å (symmetry code: (ii) (*x*, 1 - *y*, -1/2 + *z*), indicating the existence of π-π stacking between bzim ring systems of the adjacent molecules.

In the asymmetric unit two lattice water molecules locate on twofold rotation axes and are hydrogen bonded to the carboxyl groups of the complex, while the other lattice water molecule locates on a general position and links with bzim ligand of the complex *via* N—H···O hydrogen bonding (Table 2). Lattice water molecules are linked together *via* O—H···O hydrogen bonding and filled in the cavity formed by the polymeric complex chains (Fig. 3).

### Experimental

An ethanol solution (5 ml) of benzimidazole (0.236 g, 2 mmol) was mixed with an aqueous solution (5 ml) of manganese(II) acetate tetrahydrate (0.490 g, 2 mmol) at room temperature. The solution was refluxed for 30 min. Then an aqueous solution (8 ml) containing 1,3-benzenedicarboxylic acid (0.332 g, 2 mmol) and NaOH (0.160 g, 4 mmol) was added dropwise into the above solution. The mixture was refluxed for a further 2 h. After cooling to room temperature the solution was filtered. Single crystals of the title compound were obtained from the filtrate after 2 week.

### Refinement

Water H atoms were located in a difference Fourier map and refined as riding in their as-found relative positions, *U*_iso_(H) = 1.5*U*_eq_(O). Other H atoms were placed in calculated positions with N—H = 0.86 and C—H = 0.93 Å, and refined in riding mode with *U*_iso_(H) = 1.2*U*_eq_(N,*C*).

### Crystal data

**Table:**

[Mn(C_8_H_4_O_4_)(C_7_H_6_N_2_)(H_2_O)_2_]·2H_2_O	*F*(000) = 1688
*M**_r_* = 409.25	*D*_x_ = 1.540 Mg m^−^^3^
Orthorhombic, *P**b**c**n*	Mo *K*α radiation, λ = 0.71073 Å
Hall symbol: -P 2n 2ab	Cell parameters from 8868 reflections
*a* = 18.9148 (12) Å	θ = 3.0–25.0°
*b* = 23.7112 (16) Å	µ = 0.79 mm^−^^1^
*c* = 7.8701 (4) Å	*T* = 292 K
*V* = 3529.7 (4) Å^3^	Prism, yellow
*Z* = 8	0.24 × 0.20 × 0.18 mm

### Data collection

**Table:**

Rigaku R-AXIS RAPID IP diffractometer	4043 independent reflections
Radiation source: fine-focus sealed tube	3261 reflections with *I* > 2σ(*I*)
graphite	*R*_int_ = 0.029
ω scans	θ_max_ = 27.5°, θ_min_ = 2.8°
Absorption correction: multi-scan (*ABSCOR*; Higashi, 1995)	*h* = −24→20
*T*_min_ = 0.786, *T*_max_ = 0.880	*k* = −30→30
20574 measured reflections	*l* = −10→7

### Refinement

**Table:**

Refinement on *F*^2^	Primary atom site location: structure-invariant direct methods
Least-squares matrix: full	Secondary atom site location: difference Fourier map
*R*[*F*^2^ > 2σ(*F*^2^)] = 0.032	Hydrogen site location: inferred from neighbouring sites
*wR*(*F*^2^) = 0.091	H-atom parameters constrained
*S* = 1.04	*w* = 1/[σ^2^(*F*_o_^2^) + (0.0428*P*)^2^ + 2.5725*P*] where *P* = (*F*_o_^2^ + 2*F*_c_^2^)/3
4043 reflections	(Δ/σ)_max_ = 0.002
236 parameters	Δρ_max_ = 0.47 e Å^−^^3^
0 restraints	Δρ_min_ = −0.26 e Å^−^^3^

### Special details

**Table:**

Geometry. All e.s.d.'s (except the e.s.d. in the dihedral angle between two l.s. planes) are estimated using the full covariance matrix. The cell e.s.d.'s are taken into account individually in the estimation of e.s.d.'s in distances, angles and torsion angles; correlations between e.s.d.'s in cell parameters are only used when they are defined by crystal symmetry. An approximate (isotropic) treatment of cell e.s.d.'s is used for estimating e.s.d.'s involving l.s. planes.
Refinement. Refinement of *F*^2^ against ALL reflections. The weighted *R*-factor *wR* and goodness of fit *S* are based on *F*^2^, conventional *R*-factors *R* are based on *F*, with *F* set to zero for negative *F*^2^. The threshold expression of *F*^2^ > σ(*F*^2^) is used only for calculating *R*-factors(gt) *etc*. and is not relevant to the choice of reflections for refinement. *R*-factors based on *F*^2^ are statistically about twice as large as those based on *F*, and *R*- factors based on ALL data will be even larger.

### Fractional atomic coordinates and isotropic or equivalent isotropic displacement parameters (Å^2^)

**Table:**

	*x*	*y*	*z*	*U*_iso_*/*U*_eq_	
Mn	0.714708 (13)	0.679735 (11)	0.66543 (3)	0.01758 (9)	
N1	0.63968 (9)	0.50825 (7)	0.6050 (2)	0.0300 (4)	
H1	0.6084	0.4848	0.5691	0.036*	
N3	0.69188 (8)	0.59024 (6)	0.66113 (19)	0.0227 (3)	
O1	0.37986 (7)	0.73206 (5)	0.33053 (17)	0.0255 (3)	
O2	0.32557 (7)	0.81586 (6)	0.33551 (17)	0.0278 (3)	
O3	0.60704 (7)	0.71200 (6)	0.64217 (17)	0.0283 (3)	
O4	0.67794 (7)	0.78568 (7)	0.65720 (16)	0.0305 (3)	
O5	0.71498 (6)	0.68148 (5)	0.94037 (17)	0.0232 (3)	
H5A	0.7534	0.6897	1.0001	0.035*	
H5B	0.6793	0.6937	1.0010	0.035*	
O6	0.71830 (6)	0.69029 (5)	0.39202 (17)	0.0234 (3)	
H6A	0.7572	0.6963	0.3418	0.035*	
H6B	0.6861	0.7025	0.3247	0.035*	
O7	0.5000	0.66207 (9)	0.2500	0.0384 (5)	
H7A	0.4605	0.6869	0.2419	0.058*	
O8	0.5000	0.64354 (8)	0.7500	0.0321 (4)	
H8A	0.5364	0.6685	0.7171	0.048*	
O9	0.45942 (9)	0.57472 (7)	0.4780 (2)	0.0496 (4)	
H9A	0.4713	0.5996	0.3886	0.074*	
H9B	0.4773	0.5905	0.5737	0.074*	
C2	0.63681 (10)	0.56472 (8)	0.5930 (2)	0.0277 (4)	
H2	0.5996	0.5839	0.5417	0.033*	
C4	0.79996 (10)	0.54864 (8)	0.8052 (2)	0.0270 (4)	
H4	0.8220	0.5827	0.8297	0.032*	
C5	0.83029 (11)	0.49747 (9)	0.8501 (3)	0.0333 (5)	
H5	0.8739	0.4972	0.9049	0.040*	
C6	0.79648 (12)	0.44598 (9)	0.8146 (3)	0.0357 (5)	
H6	0.8180	0.4125	0.8482	0.043*	
C7	0.73256 (12)	0.44362 (8)	0.7318 (3)	0.0340 (5)	
H7	0.7106	0.4094	0.7077	0.041*	
C8	0.70224 (11)	0.49489 (8)	0.6857 (2)	0.0264 (4)	
C9	0.73497 (10)	0.54671 (7)	0.7219 (2)	0.0229 (4)	
C10	0.37791 (9)	0.78435 (8)	0.3620 (2)	0.0215 (4)	
C11	0.44298 (9)	0.81128 (7)	0.4375 (2)	0.0202 (3)	
C12	0.49726 (9)	0.77770 (7)	0.5012 (2)	0.0203 (3)	
H12	0.4924	0.7387	0.5003	0.024*	
C13	0.55886 (9)	0.80183 (8)	0.5661 (2)	0.0217 (4)	
C14	0.56567 (10)	0.86047 (8)	0.5684 (2)	0.0275 (4)	
H14	0.6069	0.8770	0.6095	0.033*	
C15	0.51092 (11)	0.89410 (8)	0.5092 (3)	0.0315 (4)	
H15	0.5152	0.9331	0.5134	0.038*	
C16	0.44988 (10)	0.86999 (8)	0.4437 (2)	0.0255 (4)	
H16	0.4135	0.8929	0.4038	0.031*	
C17	0.61786 (9)	0.76481 (8)	0.6275 (2)	0.0242 (4)	

### Atomic displacement parameters (Å^2^)

**Table:**

	*U*^11^	*U*^22^	*U*^33^	*U*^12^	*U*^13^	*U*^23^
Mn	0.01342 (14)	0.02063 (14)	0.01869 (15)	0.00030 (9)	0.00052 (9)	0.00094 (10)
N1	0.0311 (9)	0.0279 (8)	0.0310 (9)	−0.0081 (7)	0.0018 (7)	−0.0039 (7)
N3	0.0232 (7)	0.0235 (8)	0.0215 (8)	0.0005 (6)	0.0010 (6)	−0.0007 (6)
O1	0.0192 (6)	0.0291 (7)	0.0282 (7)	−0.0034 (5)	−0.0031 (5)	−0.0002 (5)
O2	0.0157 (6)	0.0364 (7)	0.0313 (8)	0.0021 (5)	−0.0017 (5)	0.0056 (6)
O3	0.0188 (6)	0.0350 (7)	0.0310 (7)	0.0057 (5)	0.0008 (5)	0.0066 (6)
O4	0.0143 (6)	0.0546 (9)	0.0226 (7)	−0.0021 (6)	−0.0021 (5)	0.0012 (6)
O5	0.0179 (6)	0.0317 (7)	0.0201 (6)	−0.0003 (5)	0.0006 (5)	−0.0032 (5)
O6	0.0184 (6)	0.0324 (7)	0.0195 (6)	0.0012 (5)	0.0016 (5)	0.0048 (5)
O7	0.0381 (12)	0.0359 (11)	0.0413 (13)	0.000	0.0073 (10)	0.000
O8	0.0264 (10)	0.0320 (10)	0.0379 (12)	0.000	0.0047 (9)	0.000
O9	0.0577 (11)	0.0495 (10)	0.0417 (10)	−0.0234 (8)	−0.0079 (8)	0.0003 (8)
C2	0.0271 (10)	0.0297 (10)	0.0264 (10)	−0.0010 (8)	0.0011 (8)	−0.0030 (8)
C4	0.0273 (10)	0.0285 (9)	0.0253 (10)	−0.0021 (7)	0.0028 (7)	0.0022 (7)
C5	0.0318 (11)	0.0394 (11)	0.0288 (11)	0.0076 (9)	0.0014 (8)	0.0056 (8)
C6	0.0468 (13)	0.0282 (10)	0.0321 (11)	0.0115 (9)	0.0113 (9)	0.0069 (8)
C7	0.0491 (13)	0.0219 (9)	0.0310 (11)	−0.0005 (8)	0.0116 (9)	0.0003 (8)
C8	0.0317 (10)	0.0250 (9)	0.0224 (9)	−0.0036 (7)	0.0070 (8)	−0.0010 (7)
C9	0.0271 (9)	0.0219 (8)	0.0195 (9)	0.0004 (7)	0.0056 (7)	0.0010 (7)
C10	0.0156 (8)	0.0308 (9)	0.0179 (8)	−0.0014 (6)	0.0015 (6)	0.0033 (7)
C11	0.0149 (8)	0.0288 (9)	0.0170 (8)	0.0004 (6)	0.0012 (6)	0.0001 (7)
C12	0.0172 (8)	0.0259 (8)	0.0178 (8)	0.0003 (6)	0.0022 (6)	−0.0008 (7)
C13	0.0171 (8)	0.0309 (9)	0.0171 (8)	0.0006 (7)	0.0009 (7)	−0.0002 (7)
C14	0.0213 (9)	0.0345 (10)	0.0267 (10)	−0.0058 (7)	−0.0030 (7)	−0.0035 (8)
C15	0.0311 (11)	0.0261 (9)	0.0373 (12)	−0.0019 (8)	−0.0034 (9)	−0.0019 (8)
C16	0.0214 (9)	0.0287 (9)	0.0265 (9)	0.0047 (7)	−0.0011 (7)	0.0008 (7)
C17	0.0164 (8)	0.0396 (11)	0.0165 (8)	0.0035 (7)	0.0023 (6)	0.0003 (7)

### Geometric parameters (Å, °)

**Table:**

Mn—N3	2.1657 (15)	C2—H2	0.9300
Mn—O2^i^	2.0995 (13)	C4—C5	1.388 (3)
Mn—O3	2.1832 (13)	C4—C9	1.394 (3)
Mn—O4	2.6075 (16)	C4—H4	0.9300
Mn—O5	2.1642 (14)	C5—C6	1.406 (3)
Mn—O6	2.1674 (14)	C5—H5	0.9300
N1—C2	1.343 (2)	C6—C7	1.375 (3)
N1—C8	1.380 (3)	C6—H6	0.9300
N1—H1	0.8600	C7—C8	1.392 (3)
N3—C2	1.319 (2)	C7—H7	0.9300
N3—C9	1.400 (2)	C8—C9	1.405 (3)
O1—C10	1.265 (2)	C10—C11	1.509 (2)
O2—C10	1.258 (2)	C11—C12	1.393 (2)
O3—C17	1.274 (2)	C11—C16	1.399 (3)
O4—C17	1.261 (2)	C12—C13	1.395 (2)
O5—H5A	0.8866	C12—H12	0.9300
O5—H5B	0.8758	C13—C14	1.396 (3)
O6—H6A	0.8469	C13—C17	1.500 (2)
O6—H6B	0.8576	C14—C15	1.388 (3)
O7—H7A	0.9532	C14—H14	0.9300
O8—H8A	0.9437	C15—C16	1.388 (3)
O9—H9A	0.9455	C15—H15	0.9300
O9—H9B	0.9065	C16—H16	0.9300
			
O2^i^—Mn—O5	90.01 (5)	C7—C6—H6	119.0
O2^i^—Mn—N3	104.35 (6)	C5—C6—H6	119.0
O5—Mn—N3	92.00 (5)	C6—C7—C8	116.76 (19)
O2^i^—Mn—O6	87.68 (5)	C6—C7—H7	121.6
O5—Mn—O6	172.03 (5)	C8—C7—H7	121.6
N3—Mn—O6	95.96 (5)	N1—C8—C7	132.35 (19)
O2^i^—Mn—O3	156.06 (5)	N1—C8—C9	105.70 (16)
O5—Mn—O3	94.55 (5)	C7—C8—C9	121.95 (19)
N3—Mn—O3	98.98 (6)	C4—C9—N3	130.56 (17)
O6—Mn—O3	84.58 (5)	C4—C9—C8	120.85 (17)
C2—N1—C8	107.21 (16)	N3—C9—C8	108.60 (16)
C2—N1—H1	126.4	O2—C10—O1	124.95 (16)
C8—N1—H1	126.4	O2—C10—C11	117.13 (16)
C2—N3—C9	105.11 (15)	O1—C10—C11	117.92 (15)
C2—N3—Mn	127.84 (13)	C12—C11—C16	119.20 (16)
C9—N3—Mn	126.95 (12)	C12—C11—C10	120.08 (15)
C10—O2—Mn^ii^	144.73 (12)	C16—C11—C10	120.72 (16)
C17—O3—Mn	101.66 (11)	C11—C12—C13	120.86 (16)
Mn—O5—H5A	122.4	C11—C12—H12	119.6
Mn—O5—H5B	123.3	C13—C12—H12	119.6
H5A—O5—H5B	105.7	C12—C13—C14	119.34 (16)
Mn—O6—H6A	120.7	C12—C13—C17	119.96 (16)
Mn—O6—H6B	129.0	C14—C13—C17	120.67 (16)
H6A—O6—H6B	105.8	C15—C14—C13	119.94 (17)
H9A—O9—H9B	105.8	C15—C14—H14	120.0
N3—C2—N1	113.38 (18)	C13—C14—H14	120.0
N3—C2—H2	123.3	C14—C15—C16	120.60 (18)
N1—C2—H2	123.3	C14—C15—H15	119.7
C5—C4—C9	117.11 (18)	C16—C15—H15	119.7
C5—C4—H4	121.4	C15—C16—C11	120.02 (17)
C9—C4—H4	121.4	C15—C16—H16	120.0
C4—C5—C6	121.4 (2)	C11—C16—H16	120.0
C4—C5—H5	119.3	O4—C17—O3	120.89 (17)
C6—C5—H5	119.3	O4—C17—C13	120.04 (17)
C7—C6—C5	121.98 (19)	O3—C17—C13	119.01 (16)

Symmetry codes: (i) *x*+1/2, −*y*+3/2, −*z*+1; (ii) *x*−1/2, −*y*+3/2, −*z*+1.

### Hydrogen-bond geometry (Å, °)

**Table:**

*D*—H···*A*	*D*—H	H···*A*	*D*···*A*	*D*—H···*A*
N1—H1···O9^iii^	0.86	1.94	2.795 (2)	171
O5—H5A···O4^iv^	0.89	1.89	2.7605 (18)	168
O5—H5B···O1^v^	0.88	1.96	2.8120 (18)	164
O6—H6A···O4^vi^	0.85	1.95	2.7553 (18)	159
O6—H6B···O1^vii^	0.86	1.88	2.7379 (18)	176
O7—H7A···O1	0.95	1.99	2.8844 (18)	155
O8—H8A···O3	0.94	1.79	2.7303 (18)	176
O9—H9A···O7	0.94	1.92	2.846 (2)	167
O9—H9B···O8	0.91	1.92	2.799 (2)	162

Symmetry codes: (iii) −*x*+1, −*y*+1, −*z*+1; (iv) −*x*+3/2, −*y*+3/2, *z*+1/2; (v) −*x*+1, *y*, −*z*+3/2; (vi) −*x*+3/2, −*y*+3/2, *z*−1/2; (vii) −*x*+1, *y*, −*z*+1/2.
